# Human socio-cultural evolution in light of evolutionary transitions: introduction to the theme issue

**DOI:** 10.1098/rstb.2021.0397

**Published:** 2023-03-13

**Authors:** Yohay Carmel, Ayelet Shavit, Ehud Lamm, Eörs Szathmáry

**Affiliations:** ^1^ Department of Environmental Engineering, Technion, Haifa, 32000, Israel; ^2^ Department of Interdisciplinary Studies, Tel Hai College, 12208, Israel; ^3^ Department of Humanities and Arts, Technion, 3200003, Israel; ^4^ The Cohn Institute for History and Philosophy of Science, Tel Aviv University, Tel Aviv, 69978, Israel; ^5^ Institute of Evolution, Centre for Ecological Research, 1121 Budapest, Hungary; ^6^ Parmenides Foundation, 82343 Pöcking, Germany; ^7^ Department of Plant Systematics, Ecology and Theoretical Biology, Eötvös University, Budapest 1117, Hungary

**Keywords:** evolutionary transitions in individuality, human evolution, cultural evolution

## Abstract

Human societies are no doubt complex. They are characterized by division of labour, multiple hierarchies, intricate communication networks and transport systems. These phenomena and others have led scholars to propose that human society may be, or may become, a new hierarchical level that may dominate the individual humans within it, similar to the relations between an organism and its cells, or an ant colony and its members. Recent discussions of the possibility of this major evolutionary transition in individuality (ETI) raise interesting and controversial questions that are explored in the present issue from four different complementary perspectives. (i) The general theory of ETIs. (ii) The unique aspects of cultural evolution. (iii) The evolutionary history and pre-history of humans. (iv) Specific routes of a possible human ETI. Each perspective uses different tools provided by different disciplines: biology, anthropology, cultural evolution, systems theory, psychology, economy, linguistics and philosophy of science. Altogether, this issue provides a broad and rich application of the notion of ETI to human past, present and perhaps also future evolution. It presents important case studies, new theoretical results and novel questions for future research.

This article is part of the theme issue ‘Human socio-cultural evolution in light of evolutionary transitions’.

## 1. Introduction

Understanding the unique evolution of humans has always been of great interest. Traditionally, the leading explanations of our unprecedented proliferation have focused on properties of individual humans, such as intelligence, imagination and creativity [1,2]. Recently, two complementary schools of thought have shifted the focus of explanation from the individual to a higher organization level, the society. The first school maintains that cumulative cultural evolution provided human groups with diverse behavioural and physiological adaptations that enabled the unprecedented propagation of our species; one clear example of this explanatory schema was produced by the anthropologist Joseph Henrich, found in his book *The secret of our success* [3]. The second school maintains that it is mostly the human capacity to cooperate, particularly in large anonymous groups, that explains human evolution, as remarkably illustrated in *The human swarm* by zoologist Mark Moffett [4]. Common to both schools is the notion that most aspects of human individual lives are increasingly dominated by human society, which becomes ever more complex. This focus on the societal dimension of human evolution is reflected in the intriguing suggestions by several scholars, that humans are (or have been) going through some sort of evolutionary transition in individuality (ETI) [5–12].

Evolution is characterized by a general increase in organismic complexity in certain lineages [13]. A few pioneering studies [14–18] have revealed that this increase is not linear; long periods of slow changes are interrupted by transformative leaps of increase in complexity. Such major evolutionary transitions (METs) occur in different times and contexts, via different mechanisms and systems, yet are explained by a few basic characteristics that reveal a surprising family resemblance among them all. The concept of MET refers to two different processes: the emergence of a new level of individuality (ETI) and the emergence of a new inheritance system. These processes often (but not always) coincide. The former practically means that entities that could previously reproduce independently now reproduce only within a larger and more complex type of individual (e.g. from a proto-cell to prokaryotic cell, from cellular to multi-cellular organism and from solitary to communal organisms). In our case, for explaining the evolutionary path of humans, both the emergence of cultural and linguistic inheritance systems, and the emergence of human societies immersed in global socio-technological tools and structures, are relevant and perhaps intertwined.

The METs at the centre of this special issue consist of a new hierarchical level of individuality above the level of the organism. Such organisms become incorporated in a newly emerging higher level of organization, in which they become ‘lower-level units’. The incorporation of individual cells within a multi-cellular organism is a notable example. Another example is the incorporation of individuals in a colony (such as in bees, ants, bryozoans and others). In many colonial species, there is an advanced division of labour within the colony, and often only certain members of the colony reproduce, while others contribute in tasks such as food gathering, colony defence, etc.

Human societies are no doubt complex. They are characterized by division of labour, multiple hierarchies, intricate communication networks and transport systems. These phenomena led Herbert Spencer [19] and Jacob von Uexküll *et al.* [20] to equate human society to an organism. In the following decades, this notion was debated and largely brought into disrepute ([21,22] and references therein). The last two decades, however, saw a wave of publications mentioning that human society may be, or may become, a higher hierarchical level that may dominate the individual human [5,6,8–12,23–25]. The present special issue applies the notions of ETI and MET, and related discussions, to human evolution, past, present and future, approaching these topics from four lines of enquiry, as follows.

## 2. Line A: human evolutionary transition in individuality in the context of evolutionary transitions

If humans have undergone or are undergoing an ETI, it may be social and/or cultural and/or technological, with or without complementary changes in human biology. Any study of such a human transition should build on a deep understanding of evolutionary transitions. Here, three papers address aspects that are common to ETIs in general. Keasar *et al.* [26] study the role of learning in honeybees. While many bee species are solitary, some species are eusocial, where the entire colony functions much like a single individual [27]. Keasar *et al*. [26] hypothesize that learning to exploit complex flowers would be more common in eusocial than in solitary species, and its likelihood would increase with increasing colony size and colony longevity. They construct a mathematical model to test these hypotheses, and their results are non-trivial. Griesemer & Shavit [28] address evolutionary transitions from a process perspective. They describe the change from an aggregated collection of individuals to a community. They focus on the role of coordination, cooperation and collaboration processes (the triple-C) in facilitating this transition. Closing this section is an explicit comparison between biological transitions and the hypothesized human transition in individuality, by Davison & Michod [29]. The authors examine steps in biological ETIs, and ask if and how they correspond to phases in human culture. They consider integrated groups of cultural traditions as the unit of study and examine hominin carnivory and the cultural change from Oldowan to Acheulean technology in comparison with behaviour in *Pan*. They argue that their analysis supports the hypothesis that human culture has undergone an ETI.

## 3. Line B: cultural evolution

The recently proliferating domain of cultural evolution is a major key for understanding the unique human evolution. It is sometimes argued that the role of cultural evolution in humans currently bypasses genetic evolution and weakens genetic adaptive potential [12]. Even those who may not agree with this claim would agree on the crucial role of cultural evolution in shaping the exceptional developments in human societies since the inception of our species, and possibly much earlier, and on the potential for gene–culture coevolution. In the context of a presumed ETI in humans, cultural evolution is thought to be a major mechanism that drives societal transitions. This perspective is addressed by six articles. A study by Denton *et al.* [30] opens this section with a puzzling question: given the potentially great fitness advantages of cumulative cultural evolution, why is a high degree of cumulative culture unique to humans? Building on and extending classic models in the literature, they suggest that there are multiple barriers to cultural accumulation, which may explain its rarity. Specialization has been often mentioned as a driver of ETIs. In this context, Ben-Oren *et al.* [31] discuss the role of specialization in humans. They demonstrate that while specialization can increase a population's cultural repertoire it may also make it less resilient to population bottlenecks. Dor [32] examines a specific human specialization: our linguistic capability. He suggests *two* evolutionary transitions for explaining its establishment, the first involving representational gestures and the second language. He argues that collaborative computation, a process unique to humans, clarifies the uniqueness of the human condition. Apes also communicate with others and imitate them, yet only humans move beyond collective mimesis to actively intervene in the mental computation of others, instructing others what to image and how to imagine it. Therefore, only humans build a network of distributed mental computation: a *collective imagination*. Kish Bar-On & Lamm [33] discuss the interplay of social identity and norm psychology in the evolution of human groups and suggest that the complexity and dynamism of human groups shaped unique features of norm psychology that are otherwise left unexplained. Szilágyi *et al.* [34] combine evolutionary ecology of early humans (moving from forests to savannahs) with niche construction theory, to propose a dynamic model for the origins of language, based on the idea that climate change meant a nutritional crisis for *Homo erectus* that was resolved by confrontational scavenging, a dangerous practice impossible without tight cooperation and protolingusitic communication. Lamm *et al.* [35] close this section with a paper that analyses the evolutionary affordances and constraints of population-level adaptations, referred to as distributed adaptations. Adaptations of this sort are often suggested in the context of human culture, but are under-theorized. Lamm *et al.* [35] explore different kinds of distributed adaptations that may have played roles in human evolution, in particular for navigation and colonization of new areas, and assess their relations to population size, density and bottlenecks.

## 4. Line C: historical and pre-historical perspectives on human evolutionary transition in individuality

The speculation that human society has undergone or is undergoing an ETI may be thought-provoking, but are there any concrete indications in support of such a view? Analyses of historical changes in human societal structure may shed some light on this issue. Here, this line of enquiry was taken by four studies. Townsend *et al.* [36] track changes in human society since the split from the apes. These authors explain human proliferation mostly by its unique culture niche as a formidable cooperator: within and between groups, among kin and non-kin, egalitarian and non-egalitarian, and on various group-sizes. Shavit & Sharon [37] describe the Neolithic revolution as an ETI: a transition from nomadic aggregates of hunter–gatherers to sedentary, interdependent and large agricultural communities. A substantial body of literature concerns the riddle of the agricultural revolution during the Neolithic, but the ETI perspective was largely ignored. Shavit & Sharon [37] attempt to use the 3Cs model presented above, in order to explain the cultural evolution in the context of ETIs. They explicate model advantages, yet do not shy away from its prices. Krall [38] also describes the agricultural revolution as an ETI, but she shifts the focus from culture and social structure to the economic system, and from an exclusive focus on humans to an analogy between humans and agricultural social insects. She maintains that the driving force of the evolutionary leap of humanity is the power dynamics of the material economic system. Using this perspective to analyse the current environmental crisis in light of global capitalism, Krall [38] then asks if current human culture is strong enough to control what she calls ‘the economic superorganism’, a collective life more reminiscent of social insects. Prentiss *et al.* [39] describe changes in Bering Sea societies, as reflected in the archaeological evidence. These changes, including social structure (from families to political groups, and the emergence of social ranking), labour specialization and communication, are described as components of an ETI. Closing this section, Carmel [40] attempts to find out if changes in human society over the last 12 000 years amount to an ETI. Towards this end, human societal development is documented, focusing on three aspects of society, presumably indicative of ETIs in general: population size, inseparability, and specialization.

## 5. Line D: realizations of the notion of human evolutionary transition in individuality

The notion of humans undergoing an evolutionary transition raises numerous questions. How is such a transition going to play out? What will be the nature of the higher-level organism, and how would the relations between the lower and the higher level look? Illustrating particular scenarios is difficult, since we need to grasp an intangible higher level above us; if such an entity does not exist, or is only beginning to emerge, how can we assess guesses about what it would look like? This line of enquiry is more speculative than previous lines. One concrete proposition for the nature of the higher-level organism was described by Carmel [40] in the previous chapter. Carmel [40] speculated that an entire society gradually becomes a primordial, crude and undeveloped organism. The present section adds two different scenarios of a human ETI. Andersson & Czárán [41] test a model published earlier [5], in which the higher level (the so-called ‘human superorganism’) consists of a closed group of humans together with their unique culture. The authors use the term ‘sociont’ to denote the presumed higher-level proto-organism and maintain that it emerged as soon as human bands became incorporated in tribes with distinct culture, namely earlier than 0.5 Ma, and possibly even 2 Ma. Another interesting proposition is made by Rainey [42], who moves the scene of the human ETI from the past to the future. Rainey [42] claims a higher-level organism may emerge as a symbiosis between humans and some form of artificial intelligence (AI), provided that fitness-affecting interactions in the societal setting are inherited by offspring. If so, an increasingly intimate co-dependency can be expected, not without perils. Following these alternative realizations of a human ETI, the last article in this section by McShea [43] closes the entire volume with a strong scepticism regarding the general notion of the human ETI. McShea [43] provides four reasons to believe that such a transition has not happened, is not currently on-going and is not likely to happen any time soon. We chose to close this volume with this sceptical atmosphere in order to emphasize that, captivating and exciting as this novel domain may be, it is still largely speculative and its ideas are tentative. However, evidence for and against concrete propositions is beginning to accumulate, and (hopefully) it may not be long before a clear picture emerges. Altogether, the papers in this special issue develop concepts, models and analyses that expand our understanding of the singular course of human evolution.

## Data Availability

This article has no additional data.

## References

[1] Fuentes A. 2018 How humans and apes are different, and why it matters. J. Anthropol. Res. **74**, 151-167. (10.1086/697150)

[2] Chu T. 2014 Human purpose and transhuman potential: a cosmic vision of our future evolution. Newburyport, MA: Red Wheel/Weiser.

[3] Henrich J. 2016 The secret of our success: how culture is driving human evolution, domesticating our species, and making us smarter. Princeton, NJ: Princeton University Press. See https://princetonup.degruyter.com/view/title/516762.

[4] Moffett MW. 2018 The human swarm. London, UK: Head of Zeus. See https://www.basicbooks.com/titles/mark-w-moffett/the-human-swarm/9781541617292/.

[5] Andersson C, Törnberg P. 2019 Toward a macroevolutionary theory of human evolution: the social protocell. Biol. Theory **14**, 86-102. (10.1007/s13752-018-0313-y)

[6] Davison DR, Andersson C, Michod RE, Kuhn SL. 2021 Did human culture emerge in a cultural evolutionary transition in individuality? Biol. Theory **16**, 213-236. (10.1007/s13752-021-00382-x)

[7] Gowdy J, Krall L. 2013 The ultrasocial origin of the Anthropocene. Ecol. Econ. **95**, 137-147. (10.1016/j.ecolecon.2013.08.006)

[8] Kesebir S. 2012 The superorganism account of human sociality: how and when human groups are like beehives. Pers. Social Psychol. Rev. **16**, 233-261. (10.1177/1088868311430834) 22202149

[9] Richerson PJ, Boyd R. 1999 Complex societies: the evolutionary origins of a crude superorganism. Hum. Nat. **10**, 253-289. (10.1007/s12110-999-1004-y) 26196336

[10] Stearns SC. 2007 Are we stalled part way through a major evolutionary transition from individual to group? Evolution **61**, 2275-2280. (10.1111/j.1558-5646.2007.00202.x) 17910743

[11] Szathmáry E. 2015 Toward major evolutionary transitions theory 2.0. Proc. Natl Acad. Sci. USA **112**, 10 104-10 111. (10.1073/pnas.1421398112) 25838283 PMC4547294

[12] Waring TM, Wood ZT. 2021 Long-term gene–culture coevolution and the human evolutionary transition. Proc. R. Soc. B **288**, 20210538. (10.1098/rspb.2021.0538) PMC8170228 34074122

[13] Pettersson M. 1996 Complexity and evolution. Cambridge, UK: Cambridge University Press.

[14] Buss LW. 1987 Evolution of individuality. Princeton, NJ: Princeton University Press.

[15] Jablonka E. 1994 Inheritance systems and the evolution of new levels of individuality. J. Theor. Biol. **170**, 301-309. 7996858 10.1006/jtbi.1994.1191

[16] Maynard Smith J. 1988 Evolutionary progress and the levels of selection. In Evolutionary progress (ed. MH Nitecky), pp. 219-230. Chicago, IL: University of Chicago Press.

[17] Maynard Smith J, Szathmáry E. 1995 The major transitions in evolution. Oxford, UK: Freeman.

[18] Szathmáry E, Maynard Smith J. 1995 The major evolutionary transitions. Nature **374**, 227-232. (10.1038/374227a0) 7885442

[19] Spencer H. 1882 The principles of sociology. New York, NY: D. Appleton.

[20] von Uexküll J, Mackinnon DL. 1926 Theoretical biology. London, UK: K. Paul, Trench, Trubner & Co. See http://archive.org/details/theoreticalbiolo00uexk.

[21] Mitman G. 1992 The state of nature: ecology, community, and American social thought, 1900–1950. Chicago, IL: University of Chicago Press.

[22] Rupert RD. 2011 Empirical arguments for group minds: a critical appraisal. Phil. Compass **6**, 630. (10.1111/j.1747-9991.2011.00420.x)

[23] Carmel Y, Shavit A. 2020 Operationalizing evolutionary transitions in individuality. Proc. R. Soc. B **287**, 20192805. (10.1098/rspb.2019.2805) PMC7031674 32019441

[24] Gowdy J, Krall L. 2014 Agriculture as a major evolutionary transition to human ultrasociality. J. Bioecon. **16**, 179-202.

[25] Powers ST, van Schaik CP, Lehmann L. 2016 How institutions shaped the last major evolutionary transition to large-scale human societies. Phil. Trans. R. Soc. B **371**, 20150098. (10.1098/rstb.2015.0098) 26729937 PMC4760198

[26] Keasar T, Pourtallier O, Wajnberg E. 2023 Can sociality facilitate learning of complex tasks? Lessons from bees and flowers. Phil. Trans. R. Soc. B **378**, 20210402. (10.1098/rstb.2021.0402) 36688396 PMC9869446

[27] Bourke AFG. 2011 Principles of social evolution. Oxford, UK: Oxford University Press.

[28] Griesemer J, Shavit A. 2023 Scaffolding individuality: coordination, cooperation, collaboration and community. Phil. Trans. R. Soc. B **378**, 20210398. (10.1098/rstb.2021.0398) 36688398 PMC9869437

[29] Davison DR, Michod RE. 2023 Steps to individuality in biology and culture. Phil. Trans. R. Soc. B **378**, 20210407. (10.1098/rstb.2021.0407) 36688387 PMC9869451

[30] Denton KK, Ram Y, Feldman MW. 2023 Conditions that favour cumulative cultural evolution. Phil. Trans. R. Soc. B **378**, 20210400. (10.1098/rstb.2021.0400) 36688392 PMC9869458

[31] Ben-Oren Y, Kolodny O, Creanza N. 2023 Cultural specialization as a double-edged sword: division into specialized guilds might promote cultural complexity at the cost of higher susceptibility to cultural loss. Phil. Trans. R. Soc. B **378**, 20210418. (10.1098/rstb.2021.0418) 36688386 PMC9869445

[32] Dor D. 2023 Communication for collaborative computation: two major transitions in human evolution. Phil. Trans. R. Soc. B **378**, 20210404. (10.1098/rstb.2021.0404) 36688385 PMC9869436

[33] Kish Bar-On K, Lamm E. 2023 The interplay of social identity and norm psychology in the evolution of human groups. Phil. Trans. R. Soc. B **378**, 20210412. (10.1098/rstb.2021.0412) 36688389 PMC9869443

[34] Szilágyi A, Kovács VP, Czárán T, Szathmáry E. 2023 Evolutionary ecology of language origins through confrontational scavenging. Phil. Trans. R. Soc. B **378**, 20210411. (10.1098/rstb.2021.0411) 36688391 PMC9869442

[35] Lamm E, Finkel M, Kolodny O. 2023 Human major transitions from the perspective of distributed adaptations. Phil. Trans. R. Soc. B **378**, 20210401. (10.1098/rstb.2021.0401) 36688390 PMC9869454

[36] Townsend C, Ferraro JV, Habecker H, Flinn MV. 2023 Human cooperation and evolutionary transitions in individuality. Phil. Trans. R. Soc. B **378**, 20210414. (10.1098/rstb.2021.0414) 36688393 PMC9869453

[37] Shavit A, Sharon G. 2023 Can models of evolutionary transition clarify the debates over the Neolithic Revolution? Phil. Trans. R. Soc. B **378**, 20210413. (10.1098/rstb.2021.0413) 36688395 PMC9869441

[38] Krall L. 2023 The economic superorganism in the complexity of evolution. Phil. Trans. R. Soc. B **378**, 20210417. (10.1098/rstb.2021.0417) 36688388 PMC9869435

[39] Prentiss AM, Laue C, Gjesfjeld E, Walsh MJ, Denis M, Foor TA. 2023 Evolution of the Okvik/Old Bering Sea culture of the Bering Strait as a major transition. Phil. Trans. R. Soc. B **378**, 20210415. (10.1098/rstb.2021.0415) 36688384 PMC9869439

[40] Carmel Y. 2023 Human societal development: is it an evolutionary transition in individuality? Phil. Trans. R. Soc. B **378**, 20210409. (10.1098/rstb.2021.0409) 36688399 PMC9869447

[41] Andersson C, Czárán T. 2023 The transition from animal to human culture—simulating the social protocell hypothesis. Phil. Trans. R. Soc. B **378**, 20210416. (10.1098/rstb.2021.0416) 36688383 PMC9869448

[42] Rainey PB. 2023 Major evolutionary transitions in individuality between humans and AI. Phil. Trans. R. Soc. B **378**, 20210408. (10.1098/rstb.2021.0408) 36688400 PMC9869444

[43] McShea DW. 2023 Four reasons for scepticism about a human major transition in social individuality. Phil. Trans. R. Soc. B **378**, 20210403. (10.1098/rstb.2021.0403) 36688394 PMC9869438

