# Correction to: Human socio-cultural evolution in light of evolutionary transitions: introduction to the theme issue (2022) by Carmel *et al.*

**DOI:** 10.1098/rstb.2023.0003

**Published:** 2023-04-10

**Authors:** Yohay Carmel, Ayelet Shavit, Ehud Lamm, Eörs Szathmáry

*Phil. Trans. R. Soc. B* **378**, 20210397. (Published 23 January 2023). (https://doi.org/10.1098/rstb.2021.0397)

In the original version of this article, an affiliation was omitted for author Eörs Szathmáry. The correct affiliations are shown below.

This has now been corrected on the publisher's website.

Eörs Szathmáry^5,6,7^

^5^Institute of Evolution, Centre for Ecological Research, 1121 Budapest, Hungary

^6^Parmenides Foundation, 82343 Pöcking, Germany

^7^Department of Plant Systematics, Ecology and Theoretical Biology, Eötvös University, Budapest 1117, Hungary

